# Rare variants in *IFFO1*, *DTNB, NLRC3* and *SLC22A10* associate with Alzheimer’s disease CSF profile of neuronal injury and inflammation

**DOI:** 10.1038/s41380-022-01437-6

**Published:** 2022-02-16

**Authors:** Alexander Neumann, Fahri Küçükali, Isabelle Bos, Stephanie J. B. Vos, Sebastiaan Engelborghs, Tim De Pooter, Geert Joris, Peter De Rijk, Ellen De Roeck, Magda Tsolaki, Frans Verhey, Pablo Martinez-Lage, Mikel Tainta, Giovanni Frisoni, Oliver Blin, Jill Richardson, Régis Bordet, Philip Scheltens, Julius Popp, Gwendoline Peyratout, Peter Johannsen, Lutz Frölich, Rik Vandenberghe, Yvonne Freund-Levi, Johannes Streffer, Simon Lovestone, Cristina Legido-Quigley, Mara ten Kate, Frederik Barkhof, Mojca Strazisar, Henrik Zetterberg, Lars Bertram, Pieter Jelle Visser, Christine van Broeckhoven, Kristel Sleegers, EMIF-AD study group, Alexander Neumann, Fahri Küçükali, Isabelle Bos, Stephanie J. B. Vos, Sebastiaan Engelborghs, Ellen De Roeck, Magda Tsolaki, Frans Verhey, Pablo Martinez-Lage, Mikel Tainta, Giovanni Frisoni, Oliver Blin, Jill Richardson, Régis Bordet, Philip Scheltens, Julius Popp, Gwendoline Peyratout, Peter Johannsen, Lutz Frölich, Rik Vandenberghe, Yvonne Freund-Levi, Johannes Streffer, Simon Lovestone, Cristina Legido-Quigley, Mara ten Kate, Frederik Barkhof, Henrik Zetterberg, Lars Bertram, Pieter Jelle Visser, Christine van Broeckhoven, Kristel Sleegers

**Affiliations:** 1 Complex Genetics of Alzheimer’s Disease Group, VIB Center for Molecular Neurology, VIB, Antwerp, Belgium; 2 Department of Biomedical Sciences, University of Antwerp, Antwerp, Belgium; 3 Netherlands Institute for Health Services Research, Utrecht, the Netherlands; 4 Alzheimer Centrum Limburg, Maastricht University, Maastricht, the Netherlands; 5 Department of Neurology and Memory Clinic, Universitair Ziekenhuis Brussel (UZ Brussel) and Center for Neurosciences (C4N), Vrije Universiteit Brussel (VUB), Brussels, Belgium; 6 Neuromics Support Facility, VIB Center for Molecular Neurology, VIB, Antwerp, Belgium; 7 Department of Neurology and Memory Clinic, Hospital Network Antwerp (ZNA) Middelheim and Hoge Beuken, Antwerp, Belgium; 8 1st Department of Neurology, School of Medicine, Faculty of Health Sciences, Aristotle University of Thessaloniki, Makedonia, Thessaloniki, Greece; 9 Department of Psychiatry and Neuropsychology, Maastricht University, Maastricht, the Netherlands; 10 School for Mental Health and Neuroscience, Maastricht University, Maastricht, the Netherlands; 11 Center for Research and Advanced Therapies, CITA—Alzheimer Foundation, San Sebastian, Spain; 12 Department of Psychiatry, Faculty of Medicine, Geneva University Hospitals, Geneva, Switzerland; 13 RCCS Instituto Centro San Giovanni di Dio Fatebenefratelli, Brescia, Italy; 14 Clinical Pharmacology & Pharmacovigilance Department, Marseille University Hospital, Marseille, France; 15 Neurosciences Therapeutic Area, GlaxoSmithKline R&D, Stevanage, UK; 16 Neuroscience & Cognition, CHU de Lille, University of Lille, Inserm, France; 17 Alzheimer Center and Department of Neurology, VU University Medical Center, Amsterdam, the Netherlands; 18 Department of Geriatric Psychiatry, University Hospital of Psychiatry Zürich, Zürich, Switzerland; 19 Old Age Psychiatry, Department of Psychiatry, University Hospital of Lausanne, Lausanne, Switzerland; 20 Department of Psychiatry, University Hospital of Lausanne, Lausanne, Switzerland; 21 Clinical Drug Development, Novo Nordisk, Copenhagen, Denmark; 22 Department of Geriatric Psychiatry, Central Institute of Mental Health, Medical Faculty Mannheim, University of Heidelberg, Mannheim, Germany; 23 Laboratory for Cognitive Neurology, Department of Neurosciences, KU Leuven, Leuven, Belgium; 24 Center for Alzheimer Research, Division of Clinical Geriatrics, Department of Neurobiology, Care Sciences and Society Karolinska Institute Stockholm Sweden, Stockholm, Sweden; 25 School of Medical Sciences Örebro, University Örebro, Örebro, Sweden; 26 Department of Psychiatry, University of Oxford, Oxford, UK; 27 Janssen Medical Ltd, High Wycombe, UK; 28 Steno Diabetes Center, Copenhagen, Denmark; 29 Institute of Pharmaceutical Sciences, King’s College London, London, UK; 30 Department of Radiology and Nuclear Medicine, VU University Medical Center, Amsterdam, the Netherlands; 31 Institutes of Neurology and Healthcare Engineering, University College London, London, UK; 32 Department of Psychiatry and Neurochemistry, University of Gothenburg, Gothenburg, Sweden; 33 Department of Molecular Neuroscience, UCL Institute of Neurology, London, UK; 34 Clinical Neurochemistry Laboratory, Sahlgrenska University Hospital, Mölndal, Sweden; 35 UK Dementia Research Institute, University College London, London, UK; 36 Hong Kong Center for Neurodegenerative Diseases, Hong Kong, China; 37 Lübeck Interdisciplinary Platform for Genome Analytics, University of Lübeck, Lübeck, Germany; 38 Centre for Lifespan Changes in Brain and Cognition, University of Oslo, Oslo, Norway; 39 Neurodegenerative Brain Diseases Group, VIB Center for Molecular Neurology, VIB, Antwerp, Belgium; 40 Complex Genetics of Alzheimer’s Disease Group, VIB Center for Molecular Neurology, VIB, Antwerp, Belgium; 41 Department of Biomedical Sciences, University of Antwerp, Antwerp, Belgium; 42 Netherlands Institute for Health Services Research, Utrecht, the Netherlands; 43 Alzheimer Centrum Limburg, Maastricht University, Maastricht, the Netherlands; 44 Department of Neurology and Memory Clinic, Universitair Ziekenhuis Brussel (UZ Brussel) and Center for Neurosciences (C4N), Vrije Universiteit Brussel (VUB), Brussels, Belgium; 45 Department of Neurology and Memory Clinic, Hospital Network Antwerp (ZNA) Middelheim and Hoge Beuken, Antwerp, Belgium; 46 1st Department of Neurology, School of Medicine, Faculty of Health Sciences, Aristotle University of Thessaloniki, Makedonia, Thessaloniki, Greece; 47 Department of Psychiatry and Neuropsychology, Maastricht University, Maastricht, the Netherlands; 48 School for Mental Health and Neuroscience, Maastricht University, Maastricht, the Netherlands; 49 Center for Research and Advanced Therapies, CITA—Alzheimer Foundation, San Sebastian, Spain; 50 Department of Psychiatry, Faculty of Medicine, Geneva University Hospitals, Geneva, Switzerland; 51 RCCS Instituto Centro San Giovanni di Dio Fatebenefratelli, Brescia, Italy; 52 Clinical Pharmacology & Pharmacovigilance Department, Marseille University Hospital, Marseille, France; 53 Neurosciences Therapeutic Area, GlaxoSmithKline R&D, Stevanage, UK; 54 Neuroscience & Cognition, CHU de Lille, University of Lille, Inserm, France; 55 Alzheimer Center and Department of Neurology, VU University Medical Center, Amsterdam, the Netherlands; 56 Department of Geriatric Psychiatry, University Hospital of Psychiatry Zürich, Zürich, Switzerland; 57 Old Age Psychiatry, Department of Psychiatry, University Hospital of Lausanne, Lausanne, Switzerland; 58 Department of Psychiatry, University Hospital of Lausanne, Lausanne, Switzerland; 59 Clinical Drug Development, Novo Nordisk, Copenhagen, Denmark; 60 Department of Geriatric Psychiatry, Central Institute of Mental Health, Medical Faculty Mannheim, University of Heidelberg, Mannheim, Germany; 61 Laboratory for Cognitive Neurology, Department of Neurosciences, KU Leuven, Leuven, Belgium; 62 Center for Alzheimer Research, Division of Clinical Geriatrics, Department of Neurobiology, Care Sciences and Society Karolinska Institute Stockholm Sweden, Stockholm, Sweden; 63 School of Medical Sciences Örebro, University Örebro, Örebro, Sweden; 64 Department of Psychiatry, University of Oxford, Oxford, UK; 65 Janssen Medical Ltd, High Wycombe, UK; 66 Steno Diabetes Center, Copenhagen, Denmark; 67 Institute of Pharmaceutical Sciences, King’s College London, London, UK; 68 Department of Radiology and Nuclear Medicine, VU University Medical Center, Amsterdam, the Netherlands; 69 Institutes of Neurology and Healthcare Engineering, University College London, London, UK; 70 Department of Psychiatry and Neurochemistry, University of Gothenburg, Gothenburg, Sweden; 71 Department of Molecular Neuroscience, UCL Institute of Neurology, London, UK; 72 Clinical Neurochemistry Laboratory, Sahlgrenska University Hospital, Mölndal, Sweden; 73 UK Dementia Research Institute, University College London, London, UK; 74 Hong Kong Center for Neurodegenerative Diseases, Hong Kong, China; 75 Lübeck Interdisciplinary Platform for Genome Analytics, University of Lübeck, Lübeck, Germany; 76 Centre for Lifespan Changes in Brain and Cognition, University of Oslo, Oslo, Norway; 77 Neurodegenerative Brain Diseases Group, VIB Center for Molecular Neurology, VIB, Antwerp, Belgium

**Keywords:** Genetics, Diagnostic markers

## Abstract

Alzheimer’s disease (AD) biomarkers represent several neurodegenerative processes, such as synaptic dysfunction, neuronal inflammation and injury, as well as amyloid pathology. We performed an exome-wide rare variant analysis of six AD biomarkers (β-amyloid, total/phosphorylated tau, NfL, YKL-40, and Neurogranin) to discover genes associated with these markers. Genetic and biomarker information was available for 480 participants from two studies: EMIF-AD and ADNI. We applied a principal component (PC) analysis to derive biomarkers combinations, which represent statistically independent biological processes. We then tested whether rare variants in 9576 protein-coding genes associate with these PCs using a Meta-SKAT test. We also tested whether the PCs are intermediary to gene effects on AD symptoms with a SMUT test. One PC loaded on NfL and YKL-40, indicators of neuronal injury and inflammation. Four genes were associated with this PC: *IFFO1*, *DTNB*, *NLRC3,* and *SLC22A10*. Mediation tests suggest, that these genes also affect dementia symptoms via inflammation/injury. We also observed an association between a PC loading on Neurogranin, a marker for synaptic functioning, with *GABBR2* and *CASZ1*, but no mediation effects. The results suggest that rare variants in *IFFO1*, *DTNB*, *NLRC3,* and *SLC22A10* heighten susceptibility to neuronal injury and inflammation, potentially by altering cytoskeleton structure and immune activity disinhibition, resulting in an elevated dementia risk. *GABBR2* and *CASZ1* were associated with synaptic functioning, but mediation analyses suggest that the effect of these two genes on synaptic functioning is not consequential for AD development.

## Introduction

Alzheimer’s disease (AD) is a neurodegenerative disease with an estimated heritability of 63% [1], with common variants explaining 9–31% of disease liability [2]. Several studies have also found a contribution of rare variants in genes such as *TREM2* and *ABCA7* toward AD [3–5]. A popular approach to discover AD relevant rare variants is the use of whole-exome/genome sequencing to assess rare-variants globally, and then to associate each variant with AD status [6]. However, the occurrence of AD is caused by a combination of pathways involving inflammation, cholesterol metabolism, tau pathology, endosome or ubiquitin-related functioning [7]. Individuals with the same symptoms can differ regarding the pathways contributing to their symptoms. At the same time, different AD relevant genes may act on different pathways. Studying AD status as an outcome may therefore mask genetic effects, which only affect specific pathways or patients subsets. In this study we focus on six CSF biomarkers, which reflect different AD relevant disease processes. The examined biomarkers are amyloid beta peptide 42 (Aβ), tau, phosphorylated tau (pTau), neurofilament light chain (NfL), YKL-40 and Neurogranin (Ng). The application of these proteins/peptides has been reviewed previously [8, 9] and is summarized below:

Aβ and tau are the two most well established AD biomarkers that reflect the defining neuropathological hallmarks of AD (amyloid plaques and tau tangles) [8, 9]. Plaque deposits of Aβ in extracellular space are one of the earliest disease processes, with accumulation beginning many years before first symptoms emerge [10]. Aβ CSF levels are inversely related to brain levels, i.e., lower levels of the 42 amino acid-long aggregation-prone Aβ in CSF are indicative of higher brain deposition of the protein [11]. pTau is one of the components of neurofibrillary tangles. Both total and pTau are increased in the CSF of AD patients [11]. Compared to Aβ, it is a more concurrent state marker of neurodegeneration, with elevated levels occurring later during disease progression [9]. NfL is a building block of axons and higher levels in CSF are indicative of neuronal injury [12]. Levels of NfL are elevated in AD patients, but it is not a specific marker of AD [9]. Another non-specific biomarker is YKL-40, which represents astrocytic activation and neuronal inflammation. YKL-40 is associated with AD status and other neuropathologies [11, 13]. Finally, neurogranin is a postsynaptic protein related to synaptic functioning, cognition and plasticity. Importantly, levels are higher in AD patients and other dementias [14].

Most genome-wide analyses use a case-control design, but some have also examined CSF and plasma biomarkers. A recent genome-wide association study (GWAS) has examined common variation in relation to Aβ and tau levels in CSF, identifying novel associations between *ZFHX3* and CSF-Aβ38 and Aβ40 levels, and confirmed a previously described sex-specific association between SNPs in *GMNC* and CSF-tTau [15]. A more recent GWAS on these datasets further identified common-variant associations between *TMEM106B* and CSF-NfL and *CPOX* and CSF-YKL-40 [16]. Another study investigated rare variants underlying plasma Aβ using whole-exome sequencing, identifying several exome-wide significant genes [17].

With this study, we took a pathway approach by analyzing rare variants in relation to distinct AD-related pathologic processes reflected by six different CSF biomarkers. As we are mainly interested in the genetics of the underlying disease processes, which can be represented by multiple biomarkers, as opposed to the biology of single biomarkers per se, we apply a multivariate approach to analyze multiple biomarkers jointly. Specifically, we applied a principal component analysis (PCA) to identify independent clusters of biomarkers representing different biological processes. A PCA approach is not only conceptually appealing, but may also improve power [18, 19].

To further improve power and generalizability, we performed a mega-analysis of two multi-center studies: the European Medical Information Framework for Alzheimer’s Disease Multimodal Biomarker Discovery (EMIF-AD MBD) study [20] and the Alzheimer’s Disease Neuroimaging Initiative (ADNI) [21].

## Methods

### Participants

This study was embedded in the EMIF-AD MBD project, a consortium of European cohort studies with the aim to increase understanding of AD pathophysiology and discover diagnostic and prognostic biomarkers [20]. The EMIF-AD MBD study includes participants with no cognitive impairment, mild cognitive impairment (MCI) or AD. Extensive phenotype information is available on diagnosis, cognition, CSF, and imaging biomarkers. Genetic assessments include genome-wide SNP and DNA methylation array data, as well as whole-exome sequencing. Written informed consent for use of data, samples and scans was obtained from all participants before inclusion in EMIF-AD MBD. The Ethical Committee of the University of Antwerp, as well as committees at each site [20], approved the study and research was in accordance with the Declaration of Helsinki.

We further included ADNI to increase power and generalizability [21]. Data used in the preparation of this article were obtained from adni.loni.usc.edu. ADNI was launched in 2003 as a public-private partnership, led by Principal Investigator Michael W. Weiner, MD. The primary goal of ADNI has been to test whether serial magnetic resonance imaging, positron emission tomography, other biological markers, and clinical and neuropsychological assessment can be combined to measure the progression of MCI and early AD.

For the main analysis we selected participants, who were assessed with exome-wide sequencing, had no known pathogenic mutations, were unrelated and had information on at most one CSF biomarker missing, resulting in a total sample size of 480 participants. Participants had mostly European ancestry (98.8%). Primary analyses were based on this multi-ancestry sample, but European ancestry only analyses are provided as sensitivity analysis (Supplementary Methods).

### Measures

#### Genotyping

Whole exome-sequencing in EMIF was performed using an Illumina NextSeq500 platform using paired-end reads on DNA samples hybridized with SeqCap EZ Human Exome Kit v3.0 (Roche). In ADNI whole exome-sequencing was performed using the Illumina HiSeq2000 platform [22] The same quality control pipeline was then applied to both studies (Supplementary Methods). Post-analysis, we retained only genes with at least two rare variant carriers in each study to reduce Type-1 error, increase generalizability and ensure convergence.

#### CSF biomarkers and dementia symptoms

CSF has been obtained via lumbar puncture and biomarker levels analyzed as previously described [21, 23]. In brief, in EMIF the V-PLEX Plus AbPeptidePanel 1 Kit assessed Aβ and INNOTEST ELISA was used for tau [23]. In ADNI the Elecsys CSF immunoassay with a cobas e 601 analyzer was used to measure Aβ and tau concentration [24]. In both EMIF and ADNI NfL was analyzed using ELISA [23, 25]. Ng was assessed using an immunoassay in EMIF [23] and electrochemiluminescence technology in ADNI [26]. YKL-40 was measured with an ELISA kit in EMIF [23] and LC/MRM‐MS proteomics in ADNI [27]. As the ADNI proteomics data contained two peptide sequences, with two ion frequencies each, we averaged across these four values after *z*-score standardization. Both studies used the Mini-Mental State Examination, a 30 item questionnaire to assess dementia symptoms [28].

### Statistical analysis

#### PCA

We first performed a PCA across both studies to identify and compute independent components using linear combinations of the measured biomarkers. Biomarkers showed extreme skewness, which can distort findings [29]. We therefore transformed all biomarkers with rank based inverse normal transformation within each cohort. The resulting z-score also harmonizes the scale between the cohorts. We used a PCA-based imputation approach, as implemented in missMDA, to account for missing levels of biomarkers [30]. To determine the optimum number of dimensions, we applied leave-one-out cross-validation minimizing the squared error of prediction. The PCA was performed in the same analysis sample as the main genetic analysis, but see sensitivity analyses for results in a larger sample not restricted by genetic information (*n* = 1158). PCA scores were computed with the psych package [31]. All analyses were performed in R 4.0.3 [32].

#### Gene-based tests

We focused on rare variants with potentially large impacts on pathogenic processes. We analyzed rare protein-coding variants with a minor allele frequency below 1% in the EMIF/ADNI population and associated them with biomarker PCs. In secondary analyses, we further prioritized loss-of-function variants.

We used a SKAT-O test [33], a kernel-based method, as implemented in MetaSKAT, allowing for heterogeneous effects between studies. MetaSKAT is an extension of the original SKAT test designed for meta-analyses [34]. As individual level data was available for both studies, we performed a mega-analysis on combined datasets.

All analyses were adjusted for sex, age and genetic ancestry. In EMIF we used the first four genome-wide PCs and in ADNI the first ten, taking into account the higher population admixture. We performed analyses both with and without adjusting for diagnosis (dummy coding for MCI and AD), to avoid collider bias in case the genetic variant and PC are both independently causative of AD [35]. To characterize which specific variants drive the gene associations, we followed up gene hits with a single variant regression analysis model analogous to the SKAT analyses.

#### Mediation tests

Genes with exome-wide significance were followed up with mediation tests. The mediation models tested whether genes impact dementia via their influence on the examined neurodegenerative process. The outcome in the models were MMSE scores and the mediator was the PC showing an exome-wide significant association with the gene. MMSE scores were normalized using a previously described method [36]. Mediation tests were performed with SMUT, an intersection-union test based on SKAT [37]. We regressed outcomes on sex, age, and genetic ancestry and z-score standardized the resulting residuals within cohorts. Normalized MMSE scores were residualized jointly across cohorts using sex, age, and genetic ancestry. These residuals were also used to correlate the PCs with MMSE to better characterize the PCs using spearman correlations. We also looked up the total effect of the genes with MMSE using the same MetaSKAT model as used with the PCs.

## Results

### Demographics

Descriptive statistics are presented in Table 1. Both EMIF and ADNI represent an elderly population of comparable ages, but EMIF recruited a larger proportion of participants with AD. The included sample of ADNI concerned only participants with no or mild cognitive impairment, resulting in a higher mean score of the MMSE, indicating better performance. See Supplementary Figs. S1 and S2 for biomarkers and PC distributions per diagnosis category.

**Table 1:** Participant characteristics.

	EMIF			ADNI		
Characteristic	*n*	Mean/%	SD	*n*	Mean/%	SD
Age	353	71.23	8.72	127	74.42	6.11
Female	182	51.6%		50	39.4%	
Education (in years)	280	10.82	3.79	127	15.94	3.13
*Ancestry*						
European	353	100%	–	121	95.3%	–
African	–	–	–	5	3.9%	–
Asian	–	–	–	1	0.8%	–
*Diagnosis*						
Cognitively normal	45	12.5%	–	62	49.6%	–
Subjective cognitive impairment	22	7.10%	–	0	0%	–
Mild cognitive impairment	185	51.7%	–	64	50.4%	–
Alzheimer’s disease	101	28.7%	–	0	0%	–
Mini-mental state examination	350	25.01	4.50	127	28.20	1.63
*CSF biomarkers*						
β-Amyloid_42_ (in pg/ml)	352	295.51	181.70	127	1024.60	476.99
Tau (in pg/ml)	351	318.27	344.14	127	276.32	101.78
Phosphorylated Tau (in pg/ml)	351	53.85	32.95	127	26.44	11.45
Neurofilament light (in pg/ml)	352	1315.39	2394.16	127	1299.76	1174.46
YKL-40 (in pg/ml or z-score^a^)	353	176946.43	67909.97	63	0.00	0.85
Neurogranin (in pg/ml)	345	127.44	193.41	127	425.86	282.45

Demographic information and descriptive statistics.

*n* sample size, *SD* standard deviation.

^a^For ADNI, average values of two peptide sequences, with two ion frequencies each, were averaged after z-score standardization.

### PCA

The cross-validation informed the use of five components (Table 2). The first component loaded strongly on the tau measures and moderately on Ng and YKL-40. Given the component’s strong loading on tTau and pTau, but also at the same time moderate loading on other aspects of neurodegeneration, we interpret the component as representing tau pathology and neurodegeneration in general. This tau pathology/degeneration PC was negatively correlated with MMSE (*r* = −0.19). The second PC loaded mostly on NfL, with a moderate loading on YKL-40, thus we can interpret it as indicating neuronal injury and inflammation. This PC correlated with MMSE negatively as well (*r* = −0.22). The third component was highly specific to Aβ and had the strongest correlation with dementia symptoms in the expected positive direction (*r* = 0.28). The fourth component loaded mostly on YKL-40 with weak loadings on tTau and NfL. This component did not correlate with MMSE scores and was therefore labeled Non-AD Inflammation. The final component loaded mostly on Ng, with weak loadings on the tau measures, but again no correlation with dementia symptoms and therefore is named Non-AD Synaptic functioning

**Table 2:** PCA results.

	Tau pathology/Degeneration			Injury/Inflammation			Aβ Pathology			Non-AD Inflammation			Non-AD Synaptic functioning		
	EMIF	ADNI	Mega	EMIF	ADNI	Mega	EMIF	ADNI	Mega	EMIF	ADNI	Mega	EMIF	ADNI	Mega
*Loadings*															
Tau	0.84	0.91	0.86	0.25	0.19	0.23	−0.06	−0.04	−0.05	0.28	0.23	0.27	0.27	0.26	0.28
pTau	0.91	0.93	0.92	0.10	0.14	0.11	−0.05	−0.14	−0.07	0.16	0.20	0.17	0.26	0.23	0.26
Aβ	−0.06	−0.09	−0.07	−0.05	−0.04	−0.04	1.00	1.00	1.00	−0.05	−0.01	−0.04	0.03	0.02	0.03
NfL	0.19	0.19	0.19	0.94	0.95	0.94	−0.05	−0.04	−0.05	0.25	0.25	0.25	0.10	0.07	0.09
YKL−40	0.31	0.28	0.31	0.31	0.28	0.30	−0.06	−0.01	−0.05	0.88	0.91	0.89	0.18	0.10	0.16
Ng	0.45	0.57	0.48	0.12	0.09	0.11	0.05	0.04	0.05	0.19	0.13	0.18	0.86	0.80	0.85
R^2^	0.32	0.36	0.33	0.18	0.17	0.18	0.17	0.17	0.17	0.17	0.17	0.17	0.16	0.13	0.15
MMSE correlation	−0.21	−0.15	−0.19	−0.25	−0.15	−0.22	0.33	0.13	0.28	−0.07	−0.01	−0.05	−0.02	0.01	−0.02

R^2^ Variance explained by component.

MMSE correlation Spearman correlation between principal component and MMSE scores. MMSE scores were residualized for age, sex, and genetic ancestry.

Principal component analysis of CSF biomarkers per study (EMIF/ADNI) and across studies (Mega). Component loadings of each biomarker (first column) on five principal components (column groups two to six) are displayed in order of the component’s explained variance (R^2^). Analyses are based on 480 participants (EMIF: 353, ADNI: 127).

The PCA results were highly consistent across both studies, however, association magnitudes with MMSE differed between studies, with ADNI showing lower effect sizes. We also performed a sensitivity analysis in a larger sample not filtered for availability of genetic assessments. The same five components were identified and loadings were nearly identical, differing at most by 0.05 (Supplementary Table S1).

### Whole-exome rare variant analysis: protein-coding variants

#### Quality control

After QC, 9576 genes remained with at least two rare variant carriers per study. Exome-wide significance was therefore set at *p* = 5.2 * 10^−6^ (Bonferroni correction). Lambda was 1 or lower, suggesting that test statistics were not inflated due to population stratification or wide-spread collider bias (Supplementary Fig. S3).

#### No diagnosis adjustment

**Fig. 1:**
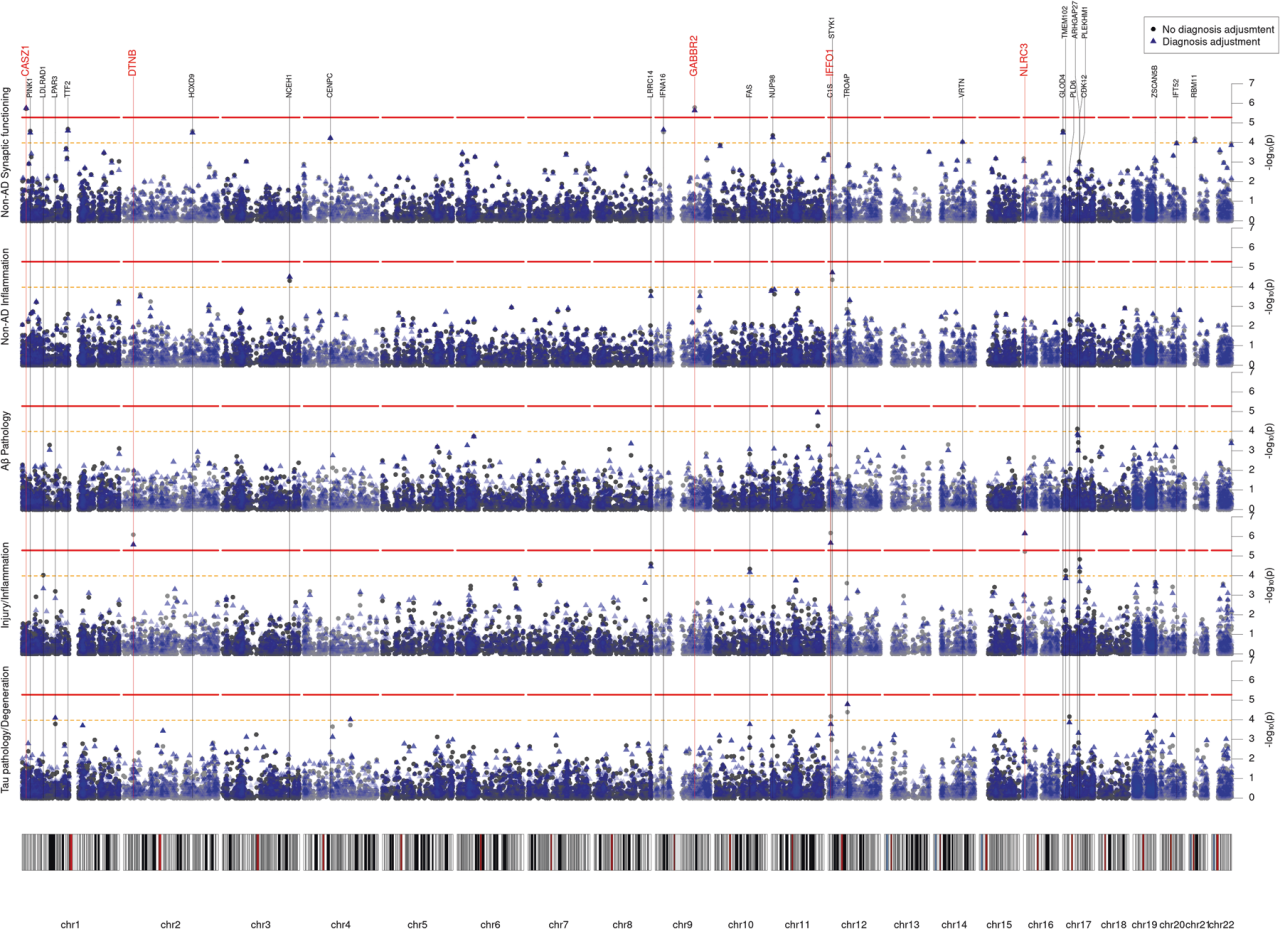
Manhattan plot of the exome-wide rare variant anayses (protein-coding). Results from the exome-wide rare variant (MAF < 1%) analyses of five CSF biomarker principal components (PC) (*n* = 480). Each plot displays a different PC as outcome. *X*-axis represents each gene (rare protein-coding variants) and the *y*-axis the *p* value obtained from gene-based SKAT-O tests on a −log_10_ scale. All analyses were adjusted for sex, age, and genetic ancestry. Blue points represent *p* values additionally adjusted for diagnosis. Red line indicates exome-wide significance threshold (*p* = 5.2 * 10^−6^). Yellow line indicates suggestive threshold (*p* = 1.0 * 10^−4^). Exome-wide significant genes are highlighted with a larger and red font.

**Fig. 2:**
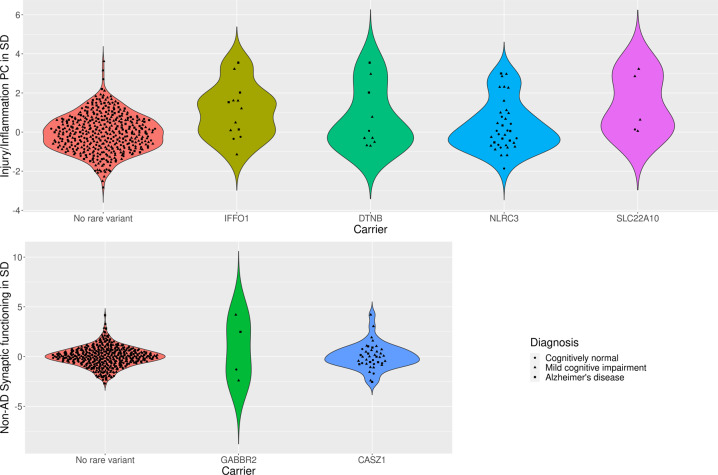
Violin plot of CSF biomarker principal component score distributions per rare variant carrier status. Top row displays the distribution of the Injury/Inflammation PC in participants not carrying a rare variant in the exome-wide significant genes, or carrying at least one variant in IFFO1, DTNB, or NLRC3. Bottom row displays the distribution of the Non-AD Synaptic functioning PC in participants not carrying a rare variant in the exome-wide significant genes, or carrying at least one variant in GABBR2, CASZ, or MICALCL. For the latter, only loss-of-function variants are considered, otherwise any protein-coding variant.

No gene passed exome-wide significance for the Tau pathology/Degeneration, Aβ Pathology, or Non-AD synaptic functioning PC. See Table 3 for gene-based results, Fig. 1 for Manhattan plots, Fig. 2 for outcome distributions per rare variant carrier status and Supplementary Table S2 for single-variant results.

**Table 3:** Genes with exome-wide significant associations.

			ADNI				EMIF				Mega					Mediation	
Outcome	Gene	Model	*n*	*n*_snps_	*n*_carriers_	*p*	*n*	*n*_snps_	*n*_carriers_	*p*	*n*	*n*_snps_	*N*_snps overlap_	*n*_carriers_	*p*	*n*	*p*
*Adjusted for sex, age and ancestry*																	
Tau pathology/Degeneration	–	–	–	–	–	–	–	–	–	–	–	–	–	–	–		
Injury/Inflammation	IFFO1	Protein	127	6	4	5.1E-01	353	4	9	2.8E-06^a^	480	8	2	13	6.7E-07^a^	478	9.5E-06^a^
	DTNB	Protein	127	20	3	5.5E-01	353	4	7	1.0E-03	480	22	2	10	8.3E-07^a^	478	1.2E-03^a^
	NLRC3	Protein	127	43	11	2.5E-01	353	23	25	6.0E-05	480	59	7	36	6.0E-06	478	2.3E-03^a^
	SLC22A10	LoF	127	4	2	7.7E-01	353	2	3	3.6E-04	480	5	1	5	5.0E-04	478	7.4E-03^a^
Aβ Pathology	–	–	–	–	–	–	–	–	–	–	–	–	–	–	–	–	–
Non-AD Inflammation	–	–	–	–	–	–	–	–	–	–	–	–	–	–	–	–	–
Non-AD Synaptic functioning	GABBR2	Protein	127	5	2	1.8E-02	353	3	2	4.3E-06^a^	480	8	0	4	1.6E-06^a^	478	8.3E-01
	CASZ1	Protein	127	43	11	3.9E-01	353	21	29	9.1E-06	480	57	7	40	1.9E-06^a^	478	8.2E-01
*Adjusted for sex, age, ancestry and diagnosis*																	
Tau pathology/Degeneration	–	–	–	–	–	–	–	–	–	–	–	–	–	–	–	–	–
Injury/Inflammation	NLRC3	Protein	127	43	11	2.8E-01	353	23	25	1.2E-05	480	59	7	36	7.0E-07^a^	–	–
	IFFO1	Protein	127	6	4	6.4E-01	353	4	9	9.1E-06	480	8	2	13	2.2E-06^a^	–	–
	DTNB	Protein	127	20	3	4.1E-01	353	4	7	1.4E-03	480	22	2	10	2.6E-06^a^	–	–
	SLC22A10	LoF	127	4	2	1	353	2	3	1.2E-04^a^	480	5	1	5	1.7E-04^a^	–	–
Aβ Pathology	–	–	–	–	–	–	–	–	–	–	–	–	–	–	–	–	–
Non-AD Inflammation	–	–	–	–	–	–	–	–	–	–	–	–	–	–	–	–	–
Non-AD Synaptic functioning	GABBR2	Protein	127	5	2	1.8E-02	353	3	2	6.3E-06	480	8	0	4	2.3E-06^a^	–	–
	CASZ1	Protein	127	43	11	4.0E-01	353	21	29	9.4E-06	480	57	7	40	1.7E-06^a^	–	–

*Model* Indicator whether variants were restricted to protein-coding (Protein) or loss-of-function (LoF) variants.

*n* Sample size.

*n*_snps_ Number of variants included.

*n*_carriers_ Number of participants with at least one rare variant in the gene.

*p* p value of SKAT-O test.

^a^Indicates exome-wide significance (protein-coding: *p* = 5.2 * 10^−6^; loss-of-function: *p* = 1.9 * 10^−4^) or nominal significance (mediation: *p* = 0.05).

*n*_snp overlap_ Number of variants present in both ADNI and EMIF.

Resuls for exome-wide rare-variant and mediation analyses. Rare (MAF < 1%) protein-coding variants in 9,576 genes were tested on a gene level in the protein-coding model and 270 genes in the loss-of-function model. Each gene was associated with five principal component scores of CSF biomarkers, representing different neurodegenerative processes. P values (*p*) were obtained from gene-based SKAT-O tests. SMUT tested mediation on dementia symptoms (MMSE scores) via changes in the principal components. All tests were adjusted for sex, age and genetic ancestry (top group). In separate models, we additionally adjusted for diagnosis status (bottom group). Only genes with exome-wide significant association in the mega-analysis (Mega) are displayed (protein-coding: *p* < 5.2 * 10^−6^; lof: *p* < 1.9 * 10^−4)^).

Two genes were associated at exome-wide significance with the Injury/Inflammation PC: *IFFO1* (*p* = 6.7 * 10^−7^) and *DTNB* (*p* = 8.3 * 10^−7^). *IFFO1* harbored five rare variants, results mostly being driven by rs138380449 and rs139792267, with five rare variant carriers each and a total of 10 carriers (Supplementary Table S2). The minor A allele in both SNPs was associated with 1.7 SD (SE = 0.44, *p* = 0.0001) and 1.3 SD (SE = 0.45, *p* = 0.0037) higher levels of injury/inflammation (Fig. 2). Both SNPs are located six bp from each other in the *IFFO1* exon. The minor alleles are missense variants resulting in a proline-to-leucine substitution, predicted to be moderately deleterious (CADD > 22.5). All carriers either had MCI (*n* = 6) or AD (*n* = 3), except one carrier with no cognitive impairment at last follow-up (age 90). Mediation tests indicated that injury/inflammation levels affected by *IFFO1* variants would also affect dementia (*p* = 9.5 * 10^−6^). For *DTNB*, rare variants were also associated with higher levels of injury/inflammation (Supplementary Table S2, Fig. 2). Mediation tests were significant as well (*p* = 0.001) and lookup of the total effect on MMSE revealed a nominally significant association (p = 0.04) (Supplementary Table S3).

Two genes also associated with the non-AD synaptic functioning PC at exome-wide significance: *GABBR2* (*p* = 1.6 * 10^−6^) and *CASZ1* (*p* = 1.9 * 10^−6^). In contrast to the Injury/Inflammation associated genes, rare variants in these genes tended to be associated with both higher and lower levels of non-AD synaptic functioning (Supplementary Table S2, Fig. 2). Given the low correlation between the non-AD synaptic functioning PC with MMSE, mediation tests were not significant (*p* ≥ 0.82).

#### Diagnosis adjustment

When adjusting for diagnosis, one additional gene reached exome-wide significance: *NLRC3* as predictor of the Injury/Inflammation PC (*p* = 7.0 * 10^−7^). As with *IFFO1* and *DTNB*, rare variants on average showed higher PC scores, e.g., the T allele in rs61732418 was associated with 0.8SD (SE = 0.41, *p* = 0.04) higher levels based on six carriers, but is likely benign (CADD = 0.1)(Supplementary Table S2, Fig. 2). As with the other Injury/Inflammation associated genes, the results suggest a mediation effect on dementia symptoms (*p* = 0.002). See Supplementary Results for sensitivity analyses (Supplementary Table S4) and single-cohort results (Supplementary Table S5).

### Whole-exome rare variant analysis: loss-of-function variants

When restricting analyses to LoF variants, 270 genes remained, which passed QC and for which at least two participants per study had rare variants. The exome-wide significance threshold was therefore set to *p* < 1.9 * 10^−4^. Most lambdas were 1.01 or lower (Supplementary Fig. S3), except for Aβ Pathology, which was 1.1 and may indicate slight inflation. One gene passed exome-wide significance in LoF prioritized models, when adjusting for diagnosis: *SLC22A10* was associated with the Injury/Inflammation PC (*p* = 1.7 * 10^−4^) (Table 3, Supplementary Fig. S4). Two rare variants in this gene associated with higher Injury/Inflammation scores, with most evidence for an A deletion in rs562147200 having deleterious effects (*β* = 1.64, SE = 0.50, *p* = 1.1 * 10^−3^) (Supplementary Table S2, Fig. 2). See Supplementary Table S6 for single-cohort results.

## Discussion

We performed the first multivariate exome-wide rare variant analysis of multiple AD CSF biomarkers in two multi-center studies. We observed a highly consistent clustering of the examined biomarkers into five independent components in both studies. We interpret the first component to represent tau pathology and neurodegeneration more generally, the second to indicate neuronal injury and inflammation, and the third component to represent Aβ pathology specifically. Not only did Aβ almost exclusively load on the third component, but Aβ also did not load on any other component. This suggests that Aβ represents a different disease process than the other biomarkers. E.g., Aβ accumulation is thought to precede first symptoms, whereas the other biomarkers are more representative of concurrent disease state [9]. The first three components correlated with dementia symptoms in the expected directions in both studies, however, the magnitude tended to be smaller in the ADNI study. The ADNI sample included only participants with MCI, resulting in higher mean MMSE scores and lower variability in the lower range, which may not generalize to clinical populations.

The fourth and fifth component loaded on YKL-40 and neurogranin, thus the first intuition may be to interpret these components as representing inflammation and synaptic functioning. However, these components did not correlate with dementia symptoms, which is at odds with previous analyses of these molecules. It is important to consider, that neurogranin and YKL-40 also loaded on the Tau/Neurodegeneration PC, and YKL-40 loaded also on the Injury/Inflammation PC. The fourth and fifth components may represent variation in inflammation and synaptic functioning, which is not related to dementia, the clinical variance being included in the first two components.

We then tested the contribution of rare variants towards the different disease processes identified by different combinations of biomarkers. *IFFO1*, *DTNB*, *NLRC3* and *SLC22A10* were associated with the Injury/Inflammation PC, as represented by heightened NfL and YKL-40 levels in the presence of rare variants in these genes. Notably, mediation tests suggested that these genes also affect dementia symptoms by impacting neural injury and inflammation. *IFFO1* codes for Intermediate filament family orphan 1 and is involved in DNA repair [38]. It is plausible that rare variants in *IFFO1* would affect NfL levels, which are themselves an intermediate filament. The results suggest that rare variants in *IFFO1* affect sensitivity of the neuronal cytoskeleton to damage, resulting in neurodegeneration and potentially dementia symptoms.

*DTNB* encodes part of the dystrophin-associated protein complex (DAPC). DAPC links actin with extracellular space, is involved in cell signaling and has been mostly studied in the context of muscle diseases [39]. Post-analysis we became aware of another simultaneously conducted study by Prokopenko et al., who performed a region-based whole genome-sequencing association analysis on AD status in an independent dataset (NIMH/NIA ADSP) [40]. Interestingly, rare variants in the *DTNB* locus were associated with AD. The converging evidence from two independent studies, using a biomarker/pathway-based approach on the one hand, and a case–control design on the other, strongly suggests an involvement of *DTNB* in neurodegenerative processes and development of AD, not previously considered.

Finally, *NLRC3* and *SLC22A10* were also associated with injury/inflammation, but only when statistically adjusting for diagnosis. However, the difference between both models was minor. *NLRC3* has a well-established role in lowering inflammation via inhibition of NfκB and NLRP3 inflammasome pathways, which have been observed to play a role in AD in human and mouse studies [41]. Specifically, downregulation of *NLRC3* in a mouse model affects plaque deposition and neuronal loss [42]. Considering that wild type *NLRC3* is involved in lowering inflammation, and overexpression has been shown to inhibit the deposition of A-beta, and reverse the degeneration of neurons in APP/PS1 mice [42], we speculate that rare variants in *NLRC3* elevate inflammation, resulting in increased neurodegeneration and dementia symptoms. SLC22A10 is an ion transporter involved in potassium homeostasis, but not much is known about its role in disease [43, 44]. According to the Agora platform, *SLC22A10* gene expression is downregulated in the parahippocampal gyrus in AD [45]. A possibility is, that the identified LoF variants in *SLC22A10* may be responsible for such downregulation and increase vulnerability to neuronal injury and inflammation.

*GABBR2* and *CASZ1* were genes identified with Non-AD synaptic functioning in protein-coding models, as mainly represented by higher levels of neurogranin. *GABBR2* encodes a GABA receptor, the main inhibitory neurotransmitter in the human brain. Downregulation of GABA receptors in various brain regions is associated with Alzheimer’s disease, potentially by disrupting the balance between excitation/inhibition balance [46, 47]. It seems plausible, that rare variants in the gene would also affect neurogranin levels and other markers of synaptic functioning. Curiously, despite prior evidence for an involvement of GABA in AD, we did not observe an association with dementia symptoms. Rare variants in the *GABBR2* gene therefore might only affect nonclinical variation of synaptic functioning, without consequences on neurodegeneration or dementia symptoms.

Finally, *CASZ1* is a zinc finger transcription factor expressed in the brain, but has been mostly studied in the context of cardiac health. For instance, LoF variants in the genes are associated with congenital heart disease [48] and cardiomyopathy [49].

Two genes reached exome-wide significance in the EMIF cohort only, but not in the mega-analysis: *CHI3L* and *CLU. CHI3L* encodes the YKL-40 protein, which is the primary biomarker loading on the non-AD inflammation PC and was recently identified as a cis-pQTL in a common-variant GWAS in an overlapping set of EMIF-AD MBD and ADNI individuals [16]. In regard to *CLU*, common and rare variants have been associated with AD and the gene product clusterin has been researched extensively as potential AD biomarker [50, 51]. Our results hint at *CLU* acting mostly via disruption of synaptic functioning, but the results have to be interpreted cautiously in light of non-replication.

The two biggest strengths of the study are the mega-analysis and multivariate design. The simultaneous analysis of multiple American and European centers and studies improves the generalizability of the results and allowed us to increase statistical power. The examination of biomarker combinations instead of single values likely supported the accurate and robust assessment of underlying disease processes, while improving power. This study is also one of the first to formally test mediation in the context of rare variant analyses using the recently developed SMUT approach.

However, as any other rare variant analysis, the chance for false positives or non-generalizable results is higher than for common variants. We opted for a mega-analysis instead of discovery-replication design to maximize robustness of initial findings. This choice also means that findings need to be externally verified before firm conclusions can be drawn. Another limitation of the study is that both CSF biomarkers and MMSE scores were measured concurrently and analyzed cross-sectionally in the mediation analyses. We can therefore not rule out reverse causation or independent pleiotropic gene effects on the biomarkers and dementia symptoms. A longitudinal analysis is recommended to explore gene effects further. More research is also needed to explore epistasis effects. Six participants carried two nominally significant risk variants across different genes, all of whom had high biomarker levels (>1.6 SD) and either a MCI or AD diagnosis. Gene-gene interaction analyses in independent samples are needed to test the potentially strong effects of carrying more than one risk gene. Finally, while the PC approach here was used to aid in etiological research, the partitioning of clinically relevant and clinically irrelevant variance for biomarkers such as YKL-40 and Ng could also improve diagnosis and prediction, which should be tested in future research.

In summary, the results suggest that rare variants in *IFFO1*, *DTNB, NLRC3, and SLC22A10* impact neuronal injury and inflammation, by potentially altering cytoskeleton structure, impairing repair abilities, and by disinhibition of immune pathways. The resulting sensitivity to damage and inflammation may then result in neurodegeneration and dementia symptoms, as evidenced by lower MMSE scores. Finally, we also found evidence for the involvement of *GABBR2 and CASZ1* in synaptic functioning, but no evidence that these changes would impact dementia symptoms.

## Supplementary information

Supplementary Methods; Results; Tables S1, S3-S6; Figures S1-S4

Table S2

Supplementary Data 1

## Data Availability

To comply with EU law and participant privacy, individual-level clinical data from EMIF-AD cannot be shared publicly, but can be requested via EMIF-AD (https://emif-catalogue.eu;http://www.emif.eu/about/emif-ad). ADNI data can be obtained from http://adni.loni.usc.edu/ after registration. See https://github.com/aneumann-science/rare_variants_csf_biomarkers for analysis code and supplementary data for full summary statistics.
